# Analysis of Personal and Functional Factors Affecting Disaster or Emergency Situation Coping Abilities in People with Intellectual Disabilities

**DOI:** 10.3390/bs16060895

**Published:** 2026-06-02

**Authors:** Eun-Young Park

**Affiliations:** Department of Secondary Special Education, Jeonju University, Jeonju 55069, Republic of Korea; eunyoung@jj.ac.kr

**Keywords:** coping abilities, disaster, emergency, functional factors, people with intellectual disabilities, personal factors

## Abstract

We aimed to explore the personal and functional factors influencing the disaster or emergency coping abilities of individuals with intellectual disabilities. To this end, this study analyzed relationships among personal factors (such as sex, age, presence of comorbid disabilities, and educational level) and functional factors (such as cognitive level and communication difficulties) using data from the disaster or emergency coping ability survey included in the 2024 Panel Survey on Work and Life of Individuals with Developmental Disabilities. In total, 1952 caregivers of individuals with intellectual disabilities responded to the survey. The analysis revealed that the level of disaster or emergency coping skills among individuals with intellectual disabilities was low. Sex, educational level, and cognitive and communication difficulties were identified as significant factors related to coping skills. Educational level was found to specifically influence the ability to evacuate oneself, a subdomain of disaster and emergency coping skills. The findings of this study suggest that systematic education and support, considering individual cognitive and communication characteristics, are necessary to improve the disaster or emergency coping abilities of individuals with intellectual disabilities.

## 1. Introduction

Modern society is facing an increasingly elevated risk of disasters because of various social and environmental factors, such as industrialization, urbanization, and climate change. Natural disasters, such as large-scale industrial accidents, earthquakes, floods, and fires, as well as social disasters, are frequently occurring worldwide, not limited to specific regions or groups, and the scale of damage is expanding (Pelling & Dill, 2010). According to the International Disaster Database of the Centre for Research on the Epidemiology of Disasters, 7348 disaster events were recorded worldwide from 2000 to 2019. These disasters killed approximately 1.23 million people—an average of 60,000 per year—and affected over 4 billion people, including those affected multiple times. They also resulted in approximately US$2.97 trillion in direct economic losses worldwide (UNDRR & CRED, 2021). These disasters not only threaten the safety of individuals and communities but also pose a major obstacle to sustainable development across society (Kelman, 2017; Noy, 2009). Accordingly, the international community has been exploring various strategies to minimize disaster risks and strengthen response capabilities.

It has been approximately 20 years since persons with disabilities were included in disaster risk reduction (DRR) policies. In 2008, the United Nations (UN) Convention on the Rights of Persons with Disabilities addressed the rights of persons with disabilities in situations of risk and humanitarian emergencies (UN, 2007). The 2013 global survey, *Living with Disability and Disasters*, conducted by the UN Office for Disaster Risk Reduction (UNDRR), went beyond simply assessing the current situation and had significant implications (UNISDR, 2014). By providing a detailed account of the disaster experiences, preparedness levels, and response challenges faced by persons with disabilities, the survey enabled the international community to recognize the need for disability-inclusive DRR more clearly. Subsequently, the Sendai Framework for Disaster Risk Reduction (2015–2030), adopted at the Third UN World Conference on Disaster Risk Reduction in 2015, emphasized ensuring the safety of vulnerable groups, particularly socially disadvantaged populations such as persons with disabilities. Moreover, it highlighted multi-stakeholder participation, strengthening disaster resilience, and institutionalizing disaster risk management as key priorities (O’Donnell, 2019; Stough & Kang, 2015).

However, in actual disaster situations, people with disabilities face significant challenges in safely evacuating or responding to emergencies. For example, the Tohoku earthquake and tsunami in Japan on 11 March 2011, demonstrated that the mortality rate among people with disabilities was four times higher than that of the general population (UNESCAP, 2014). Furthermore, a 2013 UN global survey of 5717 individuals across 126 countries found that only 20% were able to evacuate immediately, 71% had no preparedness plan, and only 31% had access to someone who could assist them during evacuation. DRR organizations are increasingly recognizing the limitations of current DRR processes in terms of disability inclusion (Robinson & Kani, 2014; Stough & Kang, 2015; World Bank & The Global Facility for Disaster Reduction and Recovery, 2017). These findings suggest that customized strategies that fully reflect the characteristics of vulnerable groups are necessary to protect all citizens from disasters.

In disability studies, the importance of the social model—which understands disability in the context of interaction with social and environmental barriers—is being emphasized, moving beyond the medical model, which understands disability solely as an individual’s functional limitation or defect (Stucki et al., 2002; WHO, 2001). From this perspective, difficulties faced by people with disabilities can be exacerbated by inaccessible environments, limited information provision systems, and non-inclusive social structures, rather than by the individual impairment itself (Barnes, 2016). In this context, criticism has been raised regarding approaches that understand the disaster vulnerability of people with disabilities merely as a result of individual disability characteristics or functional limitations. Difficulties faced by people with disabilities in responding to disasters have been argued to be closely related not only to individual functional characteristics but also to social and environmental limitations where disaster information and response systems fail to adequately consider the accessibility and comprehensibility of people with disabilities (Kailes & Enders, 2007; Stough & Kang, 2015). Kailes and Enders (2007) pointed out that existing special needs-centered approaches are overly general and fail to provide specific support strategies during actual disaster response processes, emphasizing the need for a disaster response system that addresses individual functional needs rather than classifying people with disabilities into a uniform group. Because the discomfort experienced by people vulnerable to disasters can easily recur and exacerbate potential difficulties, planning and design that reflect an understanding of disaster environments and the characteristics of vulnerable individuals are necessary from the perspective of Universal Design (Lee, 2013). Research indicates that children with disabilities can fully demonstrate their understanding of disaster risks and response capabilities when provided with appropriate support and opportunities for participation. In a participatory DRR study conducted on children with disabilities in New Zealand, Ronoh et al. (2017) reported that the children understood risks of natural disasters such as earthquakes, floods, and tsunamis, as well as protective behaviors; they were also able to distinguish between dangerous and safe zones within schools and to prioritize disaster risks. Furthermore, children with disabilities actively participated in DRR educational courses utilizing participatory tools such as mapmaking, proportional building, and survival kit activities and could offer practical opinions related to disaster response. The results of prior research suggest that the disaster vulnerability of people with disabilities should be understood in terms of shortcomings in disaster response systems and social support frameworks, rather than solely attributing it to individual defects or limitations. This research also implies that, through participation-based support strategies, people with disabilities can become active agents capable of participating in decision-making processes aimed at reducing disaster risks.

People with intellectual disabilities are particularly vulnerable to safety risks both in disasters and in everyday life. Spassiani et al. (2017) found through interviews that students with mild intellectual disabilities were unable to respond immediately to hazards during an earthquake and did not understand the importance of self-protection in both indoor and outdoor settings. Moreover, they reported limited understanding of how to handle dangerous objects, such as knives and scissors, how to use safety equipment, and the importance of basic safety skills. The cognitive limitations, communication difficulties, and limited problem-solving abilities of individuals with intellectual disabilities can increase their exposure to serious risks in disaster situations (Park, 2023). These individuals tend to rely on others during disasters and may have difficulty quickly recognizing risks or independently selecting appropriate coping strategies. Kett and van Ommeren (2009) noted that people with cognitive disabilities are particularly vulnerable to abuse and premature death in crises, such as war and disasters (Park, 2023). The UN Office for Disaster Risk Reduction (UNISDR, 2015) reported that people with disabilities face greater risks than those without disabilities during disasters, particularly those with cognitive and communication impairments, who have lower evacuation rates and limited access to information. The Korea Disaster and Safety Institute (2017) reported that individuals with developmental disabilities have difficulty understanding and implementing disaster action manuals and interpreting complex information, such as warning broadcasts, guidance messages, and evacuation instructions, making independent coping difficult and increasing reliance on caregivers. Peek and Stough (2010) identified children with disabilities as one of the most vulnerable groups in disasters, noting that children with intellectual disabilities experience multiple limitations in recognizing risks, performing coping behaviors, and communicating. Because individuals with intellectual disabilities often exhibit cognitive difficulties in executive functioning, memory, and decision-making (Alloway & Alloway, 2010; Henry et al., 2010; Willner et al., 2010), they are likely to require assistance to effectively respond in emergency situations.

However, research on disaster response capabilities among individuals with intellectual disabilities remains relatively limited compared to that for other disability groups, restricting the systematic development of policies and educational support (Stough & Kang, 2015). Functional factors are a crucial component of disaster response. For example, cognitive and communication abilities can significantly influence disaster response capabilities in this population. Nevertheless, previous disaster-related research has largely focused on physical environmental or facility-related factors, with limited attention to the role of personal and functional characteristics.

Therefore, in this study, we aimed to identify personal factors (sex, age, education level, and presence of multiple disabilities) and functional factors (communication skills and cognitive abilities) that influence disaster response among individuals with intellectual disabilities. Through this, the study seeks to empirically confirm their disaster vulnerability and provide foundational data for the development of effective safety education and training programs. Furthermore, this study is expected to contribute to the establishment of a more inclusive and sustainable disaster management system by providing a policy basis for protecting the rights and ensuring the safety of disaster-vulnerable populations.

## 2. Materials and Methods

### 2.1. Data

The analytical data for this study were derived from the 2023 Survey on the Work and Lives of Persons with Developmental Disabilities conducted by the Employment Development Institute of the Korea Employment Agency for the Disabled. The survey included 3000 households (2110 individuals and 3000 guardians) with at least one person with a developmental disability aged ≥15 years who was registered with the agency as of May 2024. The survey aims to provide an accurate understanding of the living conditions, employment status, and service needs of persons with developmental disabilities.

Data from 1952 individuals with intellectual disabilities were extracted and analyzed. Stratified random sampling methods were applied based on region and disability type. Data were collected using Tablet PC-assisted personal interviewing. The Panel Survey on the Work and Lives of Persons with Developmental Disabilities is publicly available and does not include personally identifiable information in accordance with bioethics and safety regulations; therefore, this study was exempt from ethical review and approval.

Data collection for the 2023 Survey on the Work and Lives of Persons with Developmental Disabilities was conducted using the Tablet PC Aided Personal Interview (TAPI). After training, interviewers performed the main survey by visiting the households in person. The survey participants were caregivers who had a close reciprocal influence over the individuals. Caregivers responded to the questionnaire based on daily functions and characteristics of individuals with intellectual disabilities that they cared for. The questionnaire, which included content on the ability to cope with disasters and emergencies, was designed for caregiver responses; therefore, all data analyzed in this study utilized the caregiver responses. The data used in this study were not based on self-report methods targeting individuals with intellectual disabilities directly.

### 2.2. Measures

Demographic factors included sex, age, education level, and the presence of multiple disabilities. Sex was coded as 1 for male and 2 for female. Education level was recoded on a ratio scale as follows: no education, 1; elementary school, 2; middle school, 3; high school, 4; and college or higher, 5. The presence of multiple disabilities was coded as 1 (yes) and 2 (no).

Cognitive factors included word recognition and situational awareness. The question, “What is the ability or cognitive level for each item of the person you care for?” was assessed using a 3-point Likert scale. The items included (1) reading, (2) writing, (3) number concepts, (4) awareness of dates, days of the week, and time, (5) awareness of locations and places, (6) awareness of people around you, and (7) awareness of situations. The item was measured not through actual performance assessment but rather by a method in which a guardian evaluates the overall cognitive function level of an individual with intellectual disabilities based on observation and experience. Higher scores indicate better cognitive functioning. Cronbach’s alpha was 0.960 (0.958–0.962).

Communication difficulty-related factors included language comprehension, verbal expression, nonverbal understanding, and nonverbal use. Language comprehension was measured using a 4-point Likert scale with the question, “How well does the person you care for understand what others say?” Verbal expression was measured using a 5-point Likert scale with the question, “How well does the person you care for express themselves verbally?” Nonverbal understanding was measured using a 3-point Likert scale with the question, “Can the person you care for understand nonverbal expressions such as gestures, facial expressions, and intonation?” Nonverbal use was measured using a 3-point Likert scale with the question, “Can the person you care for use nonverbal expressions, such as gestures, facial expressions, and intonation when communicating?” For reliability analysis, all communication items were converted to a 5-point scale. The higher the score, the greater the difficulty in communication. Cronbach’s alpha was 0.889 (0.882–0.895).

Disaster or emergency coping abilities were assessed using a 4-point Likert scale, with 1 indicating “not at all,” 2 indicating “somewhat,” 3 indicating “mostly able,” and 4 indicating “fully able”. The items included the following four key areas: recognizing disasters and emergencies, reporting to fire departments or police stations, evacuating independently, and asking for help. Higher scores indicate better coping ability. Cronbach’s alpha was 0.963 (0.961–0.965).

### 2.3. Statistical Analysis

This study used SPSS 26.0 (IBM Corp., Armonk, NY, USA) to conduct descriptive statistics, correlation analysis, and multiple regression analysis. Descriptive statistics were used to analyze the general characteristics of the study participants, and reliability analysis was conducted to assess the internal consistency of the scales. Correlation analysis was used to identify interrelationships among key variables. Multiple regression analysis was performed to examine the effects of demographic variables and functional factors on disaster or emergency situation coping abilities.

## 3. Results

### 3.1. General Characteristics of Participants

The general characteristics of the study participants are presented in Table 1. There were more male (1148, 58.8%) than female individuals (804, 41.2%), and the sex distribution showed a statistically significant difference (χ^2^ = 60.623, *p* < 0.001). The largest age group was 20–29 years (500, 25.6%), followed by 30–39 years (373, 19.1%), 40–49 years (312, 16.0%), 50–59 years (290, 14.9%), 60–69 years (222, 11.4%), 15–19 years (204, 10.5%), and ≥70 years (51, 2.6%). Moreover, the age distribution was statistically significant (χ^2^ = 429.413, *p* < 0.001).

Regarding educational attainment, the majority (1037 respondents, 53.1%) had a high school diploma, followed by 289 (14.8%) with no formal education, 280 (14.3%) with a middle school diploma, 240 (12.3%) with an elementary school diploma, and 106 (5.4%) with a college degree or higher. The differences among groups were statistically significant (χ^2^ = 1393.610, *p* < 0.001).

Regarding the presence of comorbid disabilities, 1762 participants (90.3%) reported having comorbid disabilities, compared to 190 (9.7%) who did not, indicating a statistically significant difference (χ^2^ = 1265.975, *p* < 0.001).

### 3.2. Correlation Among Variables

The results of examining the correlations between variables are shown in Table 2. Disaster or emergency situation coping abilities was negatively correlated with age (r = −0.183, *p* < 0.001), and positively correlated with education level (r = 0.293, *p* < 0.001), presence of comorbid disabilities (r = 0.142, *p* < 0.001), and cognitive ability (r = 0.747, *p* < 0.001). Conversely, it was negatively correlated with communication ability (r = −0.700, *p* < 0.001). Additionally, education level showed a moderate correlation with age (r = −0.626, *p* < 0.001) and cognitive ability (r = 0.418, *p* < 0.001), and cognitive ability showed a strong negative correlation with communication ability (r = −0.705, *p* < 0.001).

### 3.3. Level of Disaster or Emergency Situation Coping Abilities

Table 3 presents the results of the analysis of the level of disaster or emergency situation coping abilities. The analysis showed that the ability to recognize disasters or emergencies had the highest mean (M = 2.45, standard deviation [SD] = 1.034), with an average rank of 2.68. Next, the ability to evacuate oneself had a mean of 2.40 (SD = 1.048) and an average rank of 2.59, indicating a relatively high level. Asking others for help had a mean of 2.28 (SD = 1.027) and an average rank of 2.41, while reporting to fire stations or police stations had the lowest mean (M = 2.22, SD = 1.066) and an average rank of 2.32 among the four items.

Additionally, the analysis conducted to examine differences among the four sub-items of disaster response or emergency response abilities showed χ^2^ = 603.472, *p* < 0.001, confirming that the differences among the items were statistically significant. This indicates that the level of response behavior in disasters or emergencies varies depending on the type of behavior.

### 3.4. Factors Affecting the Disaster or Safety Coping Abilities

To identify factors influencing disaster response and safety response capabilities, a multiple regression analysis was conducted. The results are shown in Table 4. The explanatory power of the model was adjusted R^2^ = 0.787, indicating that all variables accounted for approximately 78.7% of the variance in disaster response and safety response capabilities.

Specifically, sex had a negative effect (β = −0.035, t = −3.020, *p* < 0.05), while age had a positive effect (β = 0.044, t = 3.368, *p* < 0.001). Education level also had a significant positive effect (β = 0.030, t = 2.205, *p* < 0.05). Coexisting disabilities were not statistically significant predictors (β = 0.004, t = 0.344, *p* > 0.05). Cognitive ability had the strongest positive effect on disaster and safety coping ability (β = 0.453, t = 25.470, *p* < 0.001), and communication ability showed a negative effect (β = −0.386, t = −22.755, *p* < 0.001).

## 4. Discussion

This study was conducted to examine how personal factors, such as sex, age, education level, and the presence of comorbid disabilities, as well as functional factors including cognition and communication, influence the ability of individuals with intellectual disabilities to cope with disasters or emergency situations. The results indicated that overall disaster and emergency coping abilities were significantly influenced by sex, cognitive ability, and communication skills, and these factors collectively accounted for 62.4% of the variance.

The disaster and emergency coping abilities of individuals with intellectual disabilities were found to be significantly lower than those of the general population. By category, 55.7% were able to “recognize disasters or emergency situations,” 53.5% were able to “evacuate themselves,” 46.5% were able to “ask others for help,” and 43.6% were able to “report to fire or police stations.” Abilities requiring interaction with others were relatively lower than those involving independent recognition and response. Previous studies have similarly reported that individuals with intellectual disabilities demonstrate lower verbal comprehension and nonverbal expression in disaster or emergency situations (Park, 2023), as well as reduced communication and collaborative decision-making abilities (Hansson et al., 2020), which in turn limit their capacity to respond effectively with others or to follow external instructions. While the current study results demonstrated the difficulties in disaster response and low levels of disaster safety for individuals with intellectual disabilities, these findings should not be interpreted solely as vulnerabilities inherent to the disability itself. Rather, these findings should be understood as revealing structural limitations where disaster response systems and information delivery methods failed to adequately reflect functional needs and accessibility requirements, such as cognitive and communicative characteristics of individuals with intellectual disabilities. Ronoh et al. (2017) reported that children with disabilities demonstrated a high level of understanding regarding risks of natural disasters and protective behaviors and were able to identify disaster risk factors and to suggest response strategies through participatory activities. Thus, children with disabilities may possess the capacity to actively participate in DRR when provided with appropriate support and an accessible environment.

Furthermore, the findings of this study showed that men demonstrated better disaster and safety coping abilities than women and that higher levels of cognitive ability and communication skills were associated with better coping capacity. Sex differences in behavioral functioning among individuals with intellectual disabilities have rarely been examined, and longitudinal comparisons of developmental trajectories between males and females remain limited (Hodapp & Dykens, 2005). However, prevalence studies consistently report higher rates of intellectual disability among male than female individuals (Pedersen et al., 2014). In the present study, male individuals constituted a larger proportion of the sample (58.8% male vs. 41.2% female).

Previous research has consistently highlighted education as an important factor in enhancing disaster response capabilities among people with disabilities. For example, Li et al. (2024) reported a positive relationship between educational attainment and disaster response skills, while Gaskin et al. (2017) identified formal education as a key personal factor influencing climate change vulnerability and adaptive capacity. In contrast, the present study found only a weak correlation (r = 0.293) between education level and disaster response and safety response capabilities. Among the subdomains, education was significantly associated only with self-evacuation ability. Notably, although 58.6% of participants had post-secondary education or higher (compared to 41.4% with secondary education or lower), their overall disaster response capabilities remained limited. This finding diverges from prior work, such as that by Park (2023), which identified both education level and comprehensive disaster training as significant contributors to disaster response skills.

The discrepancy may be explained by differences in access to practical training and experiential learning opportunities. Prior evidence suggests that individuals with more severe disabilities tend to have lower participation rates in disaster training, which in turn reduces their ability to effectively communicate and respond during real-life emergencies, as noted by Stough et al. (2017). Although the panel data used in this study did not include information on disaster response training, the relatively low coping abilities observed—even among those with higher education—indicate that formal education alone may be insufficient. These findings underscore the importance of proactive, hands-on training programs that provide repeated, practical experiences in disaster and safety scenarios, enabling individuals with intellectual disabilities to build functional response skills beyond theoretical knowledge.

This study found that communication skills are significantly related to disaster response and safety response capabilities. Communication is one of the major factors that can either increase or decrease vulnerability to disasters, and communication problems have a negative impact on disaster preparedness and response capabilities (Coombs, 2007). Hansson et al. (2020) reported that communication impairments delay the interpretation of warnings, understanding of emergency instructions, and the speed of requesting rescue, more than doubling the risk of harm. Therefore, communication skills are necessary to enhance disaster response capabilities. Furthermore, the importance of communication skills in disaster response and safety response capabilities should be interpreted not merely as emphasizing individual communication limitations but as suggesting the need for appropriate communication support systems. For example, accessible communication support strategies—such as Augmentative and Alternative Communication (AAC), visual cues, and repetitive demonstration training—which have been reported to be effective in improving communication and reducing problematic behaviors in individuals with developmental disabilities including intellectual disabilities, are believed to contribute to enhancing disaster understanding and response capabilities (Alzrayer et al., 2014; Ganz et al., 2017; Park, 2022; Trevor et al., 2021).

Disaster response and safety response capabilities are not merely a matter of knowledge or experience but are based on a series of cognitive processes leading to situational awareness, information processing, decision-making, and behavioral selection (McLucas, 2003); in this respect, cognitive ability can be considered a key determinant of disaster response capacity. Previous studies have consistently emphasized that cognitive ability is a core factor in disaster response and safety response capabilities (Hattori et al., 2022). Particularly in disaster situations, a series of cognitive processes involving situational awareness, risk assessment, and the selection of appropriate actions are required rather than simple reactions (Endsley, 1995; Lindell & Perry, 2012), and an individual’s level of cognitive ability acts as an important variable determining the quality of the response. Hwang and Baek (2025) explained disaster response capacity using the concept of “cognitive readiness,” suggesting that cognitive elements, such as problem-solving ability, decision-making ability, situational judgment, and adaptability, are core components of disaster response. Park (2023) reported that the ability of individuals with intellectual disabilities to cope with disasters and emergencies is closely related to their functional level, noting that difficulties in understanding situations and performing appropriate responses arise particularly when there are cognitive limitations. This implies that lower cognitive ability may limit independent responses in disaster situations. This study also supports previous research. Therefore, it is necessary to repeatedly implement customized disaster education tailored to each cognitive level. Furthermore, considering the cognitive characteristics of individuals with intellectual disabilities, disaster response training needs to be provided in a repetitive and experience-oriented manner rather than through one-time information delivery. For example, simulations utilizing actual evacuation routes, visual aids based on pictures and photos, step-by-step action guidelines, role-playing, and disaster guidance materials written in simple language can help facilitate understanding and memory for individuals with intellectual disabilities. In particular, repetitive training and daily practice can play a crucial role in enhancing the likelihood of performing actions in actual disaster situations.

This study has the advantage of ensuring the representativeness of the population of people with intellectual disabilities by analyzing panel data collected through a systematic sampling process. Nevertheless, this study has several limitations. First, the Panel Survey on Work and Life of People with Developmental Disabilities utilized in this study did not provide longitudinal data, preventing a longitudinal approach such as analyzing long-term effects over time. Therefore, longitudinal analyses are necessary in future research to identify factors influencing changes in disaster response and emergency response abilities of people with intellectual disabilities. Second, as we were only able to analyze items provided in the panel data, I was unable to fully consider various relevant variables that may be related to disaster response and emergency response abilities. While the explanatory power of the independent variables in this study was high at 62.4%, this means that 37.6% of the variance remained unexplained. Therefore, future research should consider various variables, including family and community support and safety education experiences. Third, the data herein were collected from proxy reports from guardians or primary caregivers, rather than from self-reports from the individuals with intellectual disabilities themselves. Therefore, the guardians’ perceptions, expectations, and observational experiences may have influenced the responses. Furthermore, there is evidence that guardians tend to underestimate an individual’s abilities or overprotect them, which may be a factor influencing survey responses. Fourth, the cognitive factors measured in this study were based on the guardians’ subjective evaluations rather than actual performance-based assessments. Consequently, functional levels such as reading, writing, and situational awareness may not fully reflect objective cognitive abilities. Fifth, the measurement tools used in this study did not undergo sufficient validation processes regarding the specific context of disaster response and safety response situations involving individuals with intellectual disabilities. Therefore, careful interpretation is required regarding how accurately the measurement variables reflect the actual disaster response characteristics and experiences of individuals with intellectual disabilities. Thus, future research should develop and validate scales that can more appropriately reflect the disaster response and safety response characteristics of individuals with intellectual disabilities and should utilize various data collection methods in combination.

## 5. Conclusions

This study examined the impact of disaster response and safety response skills among individuals with intellectual disabilities. Among this population, those with lower cognitive and communication skills and females demonstrated lower disaster response and safety response skills. Therefore, it is necessary to develop tailored approaches and training programs that reflect individual capabilities in order to enhance disaster and safety awareness and to provide repeated opportunities for participation in disaster drills.

## Figures and Tables

**Table 1:** Participant characteristics.

Characteristics	Frequency	%	χ^2^
Sex			
Male	1148	58.8	60.623 ***
Female	804	41.2	
Age			
15–19	204	10.5	429.413 ***
20–29	500	25.6	
30–39	373	19.1	
40–49	312	16.0	
50–59	290	14.9	
60–69	222	11.4	
70≤	51	2.6	
Education level			
No education	289	14.8	1393.610 ***
Graduate elementary school	240	12.3	
Graduate middle school	280	14.3	
Graduate high school	1037	53.1	
Above graduate college	106	5.4	
Comorbid disabilities			
Yes	190	9.7	1265.975 ***
No	1762	90.3	

Note: *** *p* < 0.001.

**Table 2:** Correlation among variables in participants.

Category	Age	Education Level	Comorbid Disabilities	Cognitive Ability	Communication Difficulties
Disaster or emergency situation coping abilities	−0.183 **	0.293 **	0.142 **	0.747 **	−0.700 **
Age		−0.626 **	−0.010	−0.270 **	0.085 **
Education level			0.012	0.418 **	−0.218 **
Comorbid disabilities				−0.156 **	0.142 **
Cognitive ability					−0.705 **

Note: ** *p* < 0.001.

**Table 3:** **Mean and mean rank** of disaster or emergency situation coping abilities.

Variables	M	SD	Mean Rank	*X^2^*	*p*
Ability to recognize disasters or emergencies ^a^	2.45	1.034	2.68	603.472	<0.001
Reporting to fire stations or police stations ^b^	2.22	1.066	2.32		
Ability to evacuate oneself ^c^	2.40	1.048	2.59		
Asking others for help ^d^	2.28	1.027	2.41		

Note. M, mean; SD, standard deviation, Items with different superscript letters within moderators are significantly different at the *p* < 0.05 level.

**Table 4:** Summary of multiple regression analysis for the level of disaster or emergency situation coping abilities.

Category	Variable	B	SE	*β*	t	*p*	VIP	*Adjusted R* ^2^
Total	Sex	−0.554	0.111	−0.070	−4.989	<0.001	1.014	0.624
	Age	−0.006	0.004	−0.024	−1.336	0.182	1.668	
	Education level	0.271	0.186	0.021	1.462	0.144	1.028	
	Presence of comorbid disability	−0.032	0.066	−0.009	−0.480	0.631	1.863	
	Cognitive abilities	0.370	0.016	0.487	22.752	<0.001	2.360	
	Communication difficulties	−0.454	0.026	−0.355	−17.731	<0.001	2.064	
Ability to recognize disasters or emergencies	Sex	−0.105	0.032	−0.051	−3.289	0.001	1.014	0.539
	Age	−0.002	0.001	−0.027	−1.350	0.177	1.668	
	Education level	0.045	0.054	0.013	0.845	0.398	1.028	
	Presence of comorbid disability	−0.008	0.019	−0.009	−0.440	0.660	1.863	
	Cognitive abilities	0.084	0.005	0.426	18.000	<0.001	2.360	
	Communication difficulties	−0.120	0.007	−0.359	−16.208	<0.001	2.064	
Reporting to fire stations or police stations	Sex	−0.140	0.032	−0.065	−4.421	<0.001	1.014	0.585
	Age	−0.003	0.001	−0.048	−2.560	0.011	1.668	
	Education level	0.007	0.053	0.002	0.137	0.891	1.028	
	Presence of comorbid disability	0.008	0.019	0.008	0.426	0.670	1.863	
	Cognitive abilities	0.106	0.005	0.511	22.754	<0.001	2.360	
	Communication difficulties	−0.101	0.007	−0.289	−13.750	<0.001	2.064	
Ability to evacuate oneself	Sex	−0.150	0.032	−0.072	−4.686	<0.001	1.014	0.549
	Age	0.0004	0.001	0.001	0.029	0.977	1.668	
	Education level	0.175	0.054	0.051	3.270	0.001	1.028	
	Presence of comorbid disability	−0.022	0.019	−0.024	−1.155	0.248	1.863	
	Cognitive abilities	0.088	0.005	0.437	18.651	<0.001	2.360	
	Communication difficulties	−0.121	0.007	−0.358	−16.326	<0.001	2.064	
Asking others for help	Sex	−0.158	0.031	−0.077	−5.105	<0.001	1.014	0.568
	Age	−0.001	0.001	−0.016	−0.806	0.420	1.668	
	Education level	0.043	0.052	0.013	0.838	0.402	1.028	
	Presence of comorbid disability	−0.009	0.019	−0.010	−0.506	0.613	1.863	
	Cognitive abilities	0.092	0.005	0.466	20.350	<0.001	2.360	
	Communication difficulties	−0.113	0.007	−0.339	−15.824	<0.001	2.064	

Note: B, unstandardized coefficient; SE, standard error; β, standardized coefficient.

## Data Availability

Data can be requested at: The data can be obtained by requesting it from the following site: https://koddi.or.kr/stat/html/user/pwpn/daap/app/pwdlPnlAplyPrcd.do accessed on 4 June 2025.

## Abbreviations

The following abbreviations are used in this manuscript:

DRR	Disaster risk reduction
CRED	Centre for Research on the Epidemiology of Disasters
SD	Standard deviation
UN	United Nations
UNESCAP	United Nations Economic and Social Commission for Asia and the Pacific
UNDRR	UN Office for Disaster Risk Reduction
UNIDRR	United Nations International Strategy for Disaster Reduction
